# Supplementary material for: Multi‐dataset identification of innovative feature genes and molecular mechanisms in keratoconus
Source: J Cell Mol Med. 2024 Sep 19;28(18):e70079. doi: 10.1111/jcmm.70079 (PMC11412914; doi:10.1111/jcmm.70079)
Supplement: Supplementary file 2 — Material S2. [file JCMM-28-e70079-s002.zip › 7.GSEA analysis/2.ATOH7/KEGG_AMYOTROPHIC_LATERAL_SCLEROSIS_ALS.html]

Details for gene set KEGG\_AMYOTROPHIC\_LATERAL\_SCLEROSIS\_ALS[GSEA]

|  || Dataset | ATOH7.group.cls#h\_versus\_l |
| Phenotype | group.cls#h\_versus\_l |
| Upregulated in class | h |
| GeneSet | KEGG\_AMYOTROPHIC\_LATERAL\_SCLEROSIS\_ALS |
| Enrichment Score (ES) | 0.43211952 |
| Normalized Enrichment Score (NES) | 1.4888134 |
| Nominal p-value | 0.0513347 |
| FDR q-value | 0.2671389 |
| FWER p-Value | 0.797 |
Table: GSEA Results Summary

  

Fig 1: Enrichment plot: KEGG\_AMYOTROPHIC\_LATERAL\_SCLEROSIS\_ALS      
 Profile of the Running ES Score & Positions of GeneSet Members on the Rank Ordered List

  

| SYMBOL | TITLE | RANK IN GENE LIST | RANK METRIC SCORE | RUNNING ES | CORE ENRICHMENT || 1 | NOS1 | na | 0 | 1.649 | 0.2354 | Yes |
| 2 | PRPH | na | 604 | 0.519 | 0.2922 | Yes |
| 3 | BCL2 | na | 1098 | 0.432 | 0.3397 | Yes |
| 4 | PRPH2 | na | 1502 | 0.386 | 0.3832 | Yes |
| 5 | MAP2K6 | na | 3338 | 0.263 | 0.3678 | Yes |
| 6 | GRIN2C | na | 3488 | 0.256 | 0.4001 | Yes |
| 7 | TNFRSF1B | na | 4331 | 0.224 | 0.4078 | Yes |
| 8 | ALS2 | na | 6173 | 0.166 | 0.3784 | Yes |
| 9 | MAPK12 | na | 6613 | 0.154 | 0.3877 | Yes |
| 10 | PPP3CC | na | 7238 | 0.137 | 0.3893 | Yes |
| 11 | MAP3K5 | na | 7445 | 0.132 | 0.4022 | Yes |
| 12 | BCL2L1 | na | 7603 | 0.128 | 0.4160 | Yes |
| 13 | APAF1 | na | 7669 | 0.126 | 0.4321 | Yes |
| 14 | GRIN1 | na | 8945 | 0.100 | 0.4096 | No |
| 15 | PPP3CA | na | 11626 | 0.057 | 0.3405 | No |
| 16 | TNFRSF1A | na | 11768 | 0.055 | 0.3444 | No |
| 17 | PPP3CB | na | 11848 | 0.055 | 0.3499 | No |
| 18 | NEFL | na | 12990 | 0.041 | 0.3228 | No |
| 19 | SOD1 | na | 13340 | 0.037 | 0.3180 | No |
| 20 | CAT | na | 13546 | 0.035 | 0.3171 | No |
| 21 | RAB5A | na | 13630 | 0.034 | 0.3195 | No |
| 22 | CASP9 | na | 14196 | 0.029 | 0.3073 | No |
| 23 | TNF | na | 14399 | 0.027 | 0.3053 | No |
| 24 | CHP1 | na | 14674 | 0.024 | 0.3008 | No |
| 25 | TP53 | na | 14698 | 0.024 | 0.3035 | No |
| 26 | DERL1 | na | 15374 | 0.018 | 0.2866 | No |
| 27 | PPP3R1 | na | 15411 | 0.018 | 0.2881 | No |
| 28 | MAP2K3 | na | 15568 | 0.016 | 0.2859 | No |
| 29 | CYCS | na | 16260 | 0.011 | 0.2675 | No |
| 30 | RAC1 | na | 16331 | 0.010 | 0.2669 | No |
| 31 | BAD | na | 17218 | 0.002 | 0.2417 | No |
| 32 | DAXX | na | 17402 | 0.001 | 0.2366 | No |
| 33 | BAX | na | 17508 | 0.000 | 0.2336 | No |
| 34 | GPX1 | na | 18080 | -0.005 | 0.2178 | No |
| 35 | CCS | na | 18115 | -0.005 | 0.2175 | No |
| 36 | TOMM40 | na | 18525 | -0.009 | 0.2070 | No |
| 37 | CASP1 | na | 18648 | -0.010 | 0.2049 | No |
| 38 | GRIA1 | na | 18719 | -0.010 | 0.2043 | No |
| 39 | NEFM | na | 19108 | -0.014 | 0.1951 | No |
| 40 | BID | na | 20508 | -0.027 | 0.1586 | No |
| 41 | GRIA2 | na | 22624 | -0.050 | 0.1047 | No |
| 42 | MAPK14 | na | 23324 | -0.059 | 0.0930 | No |
| 43 | MAPK13 | na | 23983 | -0.069 | 0.0840 | No |
| 44 | TOMM40L | na | 24620 | -0.079 | 0.0769 | No |
| 45 | CASP3 | na | 24828 | -0.083 | 0.0827 | No |
| 46 | GRIN2D | na | 25022 | -0.086 | 0.0894 | No |
| 47 | MAPK11 | na | 25479 | -0.094 | 0.0897 | No |
| 48 | GRIN2B | na | 28274 | -0.158 | 0.0317 | No |
| 49 | GRIN2A | na | 28440 | -0.163 | 0.0502 | No |
| 50 | CHP2 | na | 28497 | -0.164 | 0.0720 | No |
| 51 | NEFH | na | 28953 | -0.178 | 0.0843 | No |
| 52 | SLC1A2 | na | 30764 | -0.238 | 0.0661 | No |
| 53 | PPP3R2 | na | 32636 | -0.337 | 0.0603 | No |
Table: GSEA details [plain text format]

  

Fig 2: KEGG\_AMYOTROPHIC\_LATERAL\_SCLEROSIS\_ALS      
 Blue-Pink O' Gram in the Space of the Analyzed GeneSet

  

Fig 3: KEGG\_AMYOTROPHIC\_LATERAL\_SCLEROSIS\_ALS: Random ES distribution      
 Gene set null distribution of ES for **KEGG\_AMYOTROPHIC\_LATERAL\_SCLEROSIS\_ALS**
